# Embryos resolve the enigmatic origin of the theropod wishbone

**DOI:** 10.1126/sciadv.aeh7555

**Published:** 2026-10-07

**Authors:** Sebastian Jiménez-Villalón, Felipe Guajardo-Pezo, Mackarenna Narváez, Daniel Smith-Paredes, Tiana Kohlsdorf, Joao F. Botelho, Alexander O. Vargas

**Affiliations:** ^1^ Laboratorio de Ontogenia y Filogenia, Departamento de Biología, Facultad de Ciencias, Universidad de Chile, Santiago, Chile.; ^2^ Laboratorio de Evolución y Desarrollo, Escuela de Medicina Veterinaria, Pontificia Universidad Católica de Chile, Santiago, Chile.; ^3^ Laboratorio de Histopatología. Escuela de Medicina Veterinaria, Pontificia Universidad Católica de Chile, Santiago, Chile.; ^4^ Centre for Interdisciplinary Research in Biology, Collège de France, CNRS, Paris, France.; ^5^ Department of Biology, FFCLRP, University of São Paulo. Avenida Bandeirantes, 3900, Bairro Monte Alegre. Ribeirão Preto, São Paulo, Brazil.; ^6^ Núcleo Milenio Transiciones Evolutivas Tempranas de Mamíferos, Santiago, Chile.

## Abstract

The origin of the avian wishbone (furcula)—present since theropod dinosaurs—remains unsolved. Its homology is still debated, with competing hypotheses proposing derivation from the clavicles, the interclavicle, or both. To obtain comparative developmental evidence, we studied ossification and pre-ossification mesenchymal condensations in a broad sample of birds and non-avian reptiles. Relative positions of paired condensations and their ossification centers support homology between the lateral arms of the furcula and reptilian clavicles. However, in the palaeognathous bird *Nothoprocta perdicaria*, we observed a separate interclavicle-like medial condensation. It quickly fuses with the clavicular condensations but then develops its own ossification center. The phylogenetic distribution of this developmental pattern across reptiles and birds supports a composite origin of the furcula from clavicles and the interclavicle. Early fusion of condensations leads to seamless integration, which may explain why fossil evidence of this composite origin has remained elusive.

## INTRODUCTION

The identity of the wishbone (furcula) of birds has long been the subject of debate. As early as the 15th century, Belon identified homologs of most human bones in the chicken but regarded the furcula as problematic (*1*). For most of the 20th century, the apparent lack of a clear homolog in dinosaurs was considered to exclude them from being bird ancestors (*2*, *3*). By the 1990s, however, it became clear that a small furcula was indeed present in theropod dinosaurs, although often overlooked or unpreserved (*4*, *5*). With the dinosaur-bird link no longer in question, the debate on the origin of the furcula would seemingly be over; however, it has once again been reignited by authors who question the common assumption that the theropod furcula evolved from the medial fusion of the clavicles (*3*, *6*). The alternative possibilities are that it is derived from the interclavicle, a single medial bone ancestrally present in the reptilian shoulder girdle (Fig. 1) (*7*, *8*), or that the furcula represents a composite element derived from both the clavicles and the interclavicle (*9*).

**Fig. 1.**
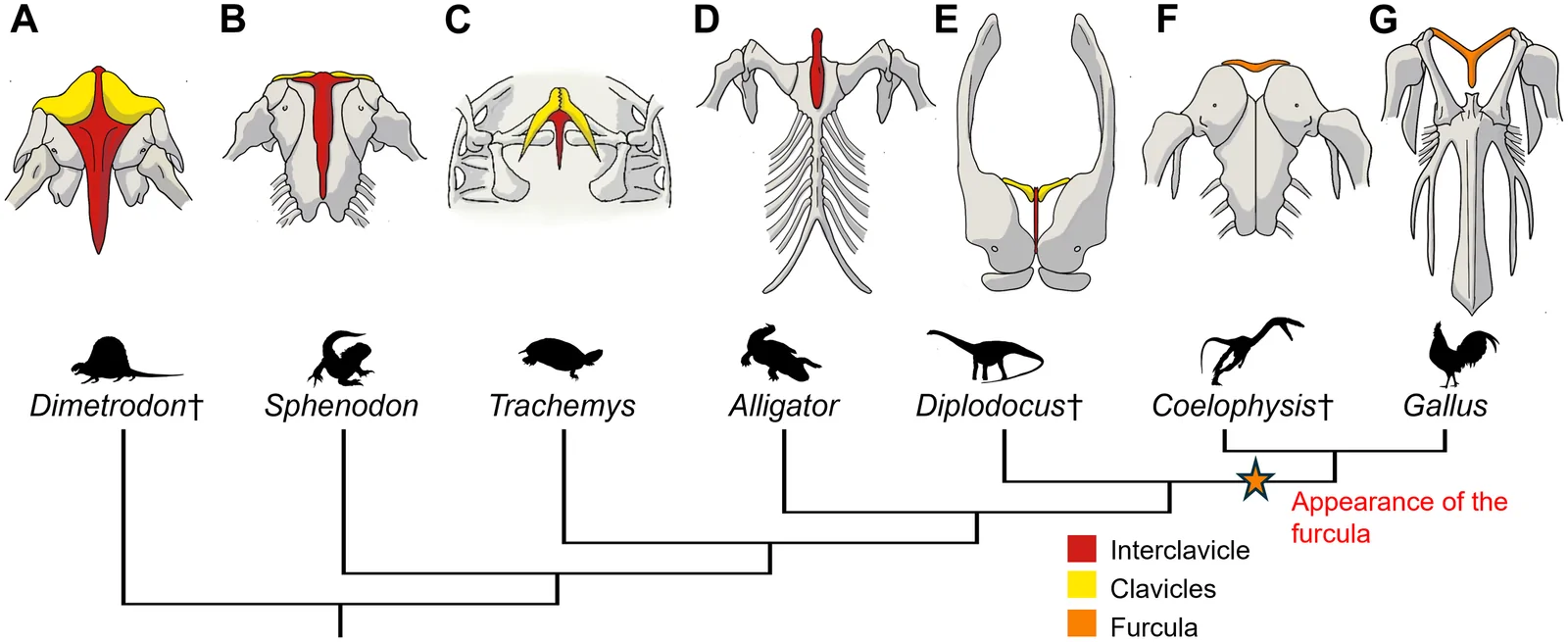
Evolution of the dermal shoulder girdle among amniotes. The pectoral apparatus of selected amniotes is shown in ventral view, highlighting dermal elements. Clavicles in yellow; interclavicles in red; furculae in orange. (**A**) In early amniotes, both clavicle and interclavicle are robust and wide. (**B**) In lepidosaurs, the interclavicle consists of a flattened longitudinal bar that may have elongated lateral processes that give it a “T” or cruciform morphology, while the clavicles are shaped like curved rods. (**C**) In turtles, the clavicles and the interclavicle are integrated into the plastron, corresponding to the paired epiplastron and the medial entoplastron respectively (*8*). (**D**) In crocodilians, the closest living relatives of birds, clavicles are absent and the interclavicle lacks lateral processes; however, their ancestors, the early pseudosuchians, still retained both elements. (**E**) In the sauropod *Diplodocus*, a pair of L-shaped clavicles and a bilaterally symmetrical interclavicle were present (*13*). (**F**) Furcula originated early in theropod evolution, before the divergence of Coelophysidae, as indicated by the star (*5*). (**G**) In modern birds, the furcula often presents a “V” morphology or a “Y” morphology when it bears a hypocleidium process as in *Gallus*.

Paired clavicles have been identified in several non-theropod dinosaurs, and both clavicles and an interclavicle are present in sauropodomorphs (*10*–*14*). However, basal theropods already exhibit a distinctive V-shaped furcula, leaving no intermediate fossil evidence for the transformation of the clavicles and/or the interclavicle into the furcula (Fig. 1) (*5*). Most paleontologists assume that the theropod furcula evolved by the medial fusion of the clavicles, but in *Halszkaraptor*, a theropod closely related to birds, the furcula is firmly connected to the sternum, resembling the joint between the interclavicle and the sternum in other reptiles, which suggests that the furcula would be comparable to the interclavicle (*7*).

Comparative developmental studies on the Alligator and Chicken show that both the interclavicle and the furcula arise from paired preosteogenic condensations that fuse at the midline, rendering the interclavicle as parsimonious as the clavicles as a potential homolog of the furcula (*8*). However, descriptions of furcular development are almost entirely restricted to galliform model species; other avian lineages have been scarcely investigated, and developmental studies of reptilian clavicles are limited (*8*, *15*, *16*). Moreover, because homology assessment relies on morphology and topology, three-dimensional (3D) analyses of preosteogenic stages are essential and still lacking for most relevant taxa.

To obtain comparative developmental evidence on furcular homology and evolution, we examined the osteogenic and preosteogenic patterns of the clavicles and interclavicle in several non-avian reptiles and of the furcula in four birds. We show that in the palaeognathous Chilean tinamou (*Nothoprocta perdicaria*), furcular development includes an independent medial ossification center and preosteogenic condensation comparable to the interclavicle, supporting a composite origin of the furcula from both clavicles and the interclavicle.

## RESULTS

### Ossification pattern of the furcula

The tetrapod shoulder girdle is a complex structure that integrates endochondral bones, which ossify from a cartilage template, and dermal bones, which ossify directly by intramembranous ossification (*8*, *17*, *18*). In birds and their theropod ancestors, the furcula is the only dermal bone of the shoulder girdle (*8*, *16*). Evolutionary patterns of skeletal development are much better understood for ossification stages than for pre-osteogenic stages; information about the latter is only available from a comparatively much smaller sample of vertebrates. In contrast, 3D information about ossification patterns can be obtained more readily, even from late-stage or adult specimens such as fossils, which are often directly informative. To understand the homology of the furcula, our first approach was therefore to compare ossification patterns across a broad sample of avian and non-avian reptiles. Questions raised or left unresolved by the ossification patterns were then pursued through targeted study of earlier pre-osteogenic stages.

We investigated the ossification of the furcula in four bird species broadly distributed across avian phylogeny and with divergent furcular morphologies: the Chilean tinamou (*N. perdicaria*), a palaeognathous bird with a complete furcula; the chicken (*Gallus gallus*), a Galloanserae with a complete furcula; and two Neoaves: the zebra finch (*Taeniopygia guttata*), with a complete furcula, and the budgerigar (*Melopsittacus undulatus*), with a reduced furcula.

The ossification pattern of the furcula of *Taeniopygia*, a passeriform, is similar to that described for galliformes (*Gallus* and *Coturnix*) (Fig. 2A) (*8*, *15*, *16*). The furcula is one of the first skeletal elements to ossify, starting only after the long bones of the limbs and coinciding with the appearance of the first ossification centers in the skull. Thin and elongated centers appear on the lateral third of each arm of the furcula and extend toward the midline until they fuse in stage Hamburger and Hamilton (HH) 35 (*19*), with the hypocleidium arising as an incipient projection from this fused center (Fig. 2, A to C).

**Fig. 2.**
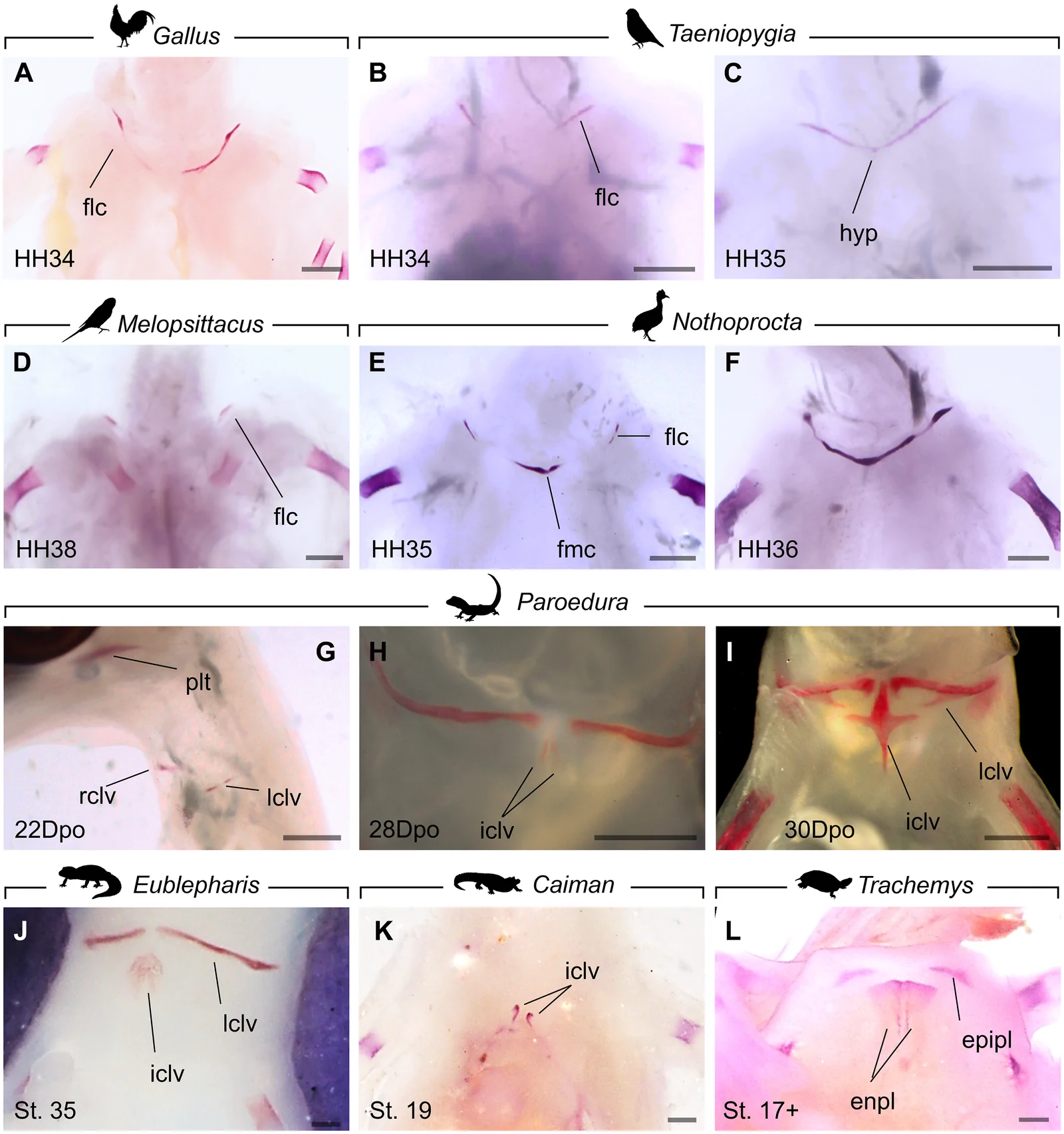
Ossification pattern of the dermal shoulder girdle. (**A** to **F**) Alizarin red staining embryos in a ventral view. (A) Lateral ossification centers of the furcula in HH34 *Gallus* and (B) in HH34 *Taeniopygia* embryos. (C) Furcula in a HH35 *Taeniopygia* embryo with an incipient hypocleidium. (D) Furcular centers in a HH38 *Melopsittacus* embryo. (E) Ossification centers in the furcula of a HH35 *Nothoprocta* embryo, showing two lateral centers, as observed in Neognathae, and an additional medial center. (F) Furcula in a HH36 *Nothoprocta* embryo. (**G** to **I**) Alizarin red staining embryos of *Paroedura*: (G) Clavicular ossification centers in a 22dpo embryo in ventrolateral view, (H) paired interclavicular center in a 28dpo embryo in ventral view, and (I) dermal shoulder girdle in a 30dpo stage embryo. (**J**) Stage 35 *Eublepharis* embryo showing long clavicular centers and cranially fused interclavicular centers. (**K**) Stage 19 *Caiman* embryo showing the paired nonfused interclavicular ossification centers. (**L**) Late stage 17 *Trachemys* embryo showing lateral paired epiplastral (clavicular) centers and cranially fused entoplastral (interclavicular) centers. Labeled anatomical elements: flc, furcular lateral center; fmc, furcular medial center; hyp, hypocleidium; iclv, interclavicle; lclv, left clavicle; rclv, right clavicle; plt, palatine; enpl, entoplastron; epipl, epiplastron. Scale bars, 1 mm.

Most parrots have reduced furculae (*20*). In adult *Melopsittacus*, the furcula comprises a pair of small and separate splinter-like arms that articulate with the acromion of the scapula (fig. S1D). During development, its ossification is delayed. Paired lateral ossification centers first appear at stage HH38, when other skeletal elements (e.g., skull, scapula, and coracoid) are already well ossified (Fig. 2D). There was no extension of ossification centers toward the midline, and no other ossification center attributable to the furcula was observed at any stage (fig. S1C).

The furcula is absent or reduced in all nonflying paleognathous birds, but it is present and well-formed as an U-shaped element in tinamiformes (*9*). In Chilean tinamou, *N. perdicaria* ossification begins at stage HH35. Three separate centers were observed at this stage: a pair of lateral centers corresponding to those observed in the other bird species studied, plus a medial center with a bilaterally symmetrical morphology of paired lobes (fmc in Fig. 2E and see also fig. S1, E and F). At stage HH36, the medial ossification center extends laterally, although there is still discontinuity with the lateral centers (Fig. 2F). The three ossification centers are completely fused at stage HH37.

Therefore, we identified three distinct ossification patterns in the furcula of modern birds: (i) bilateral ossification centers that extend toward the midline and fuse with each other (*G. gallus* and *Taeniopygia*), (ii) bilateral centers that remain separate (*Melopsittacus*), and (iii) bilateral centers that extend toward the midline and meet a third, medial ossification center (*Nothoprocta*).

### Ossification pattern of the dermal shoulder girdle in non-avian reptiles

Ancestrally, the reptilian dermal shoulder girdle included a pair of clavicles and a single medial interclavicle (Fig. 1). Crocodilians, the closest living relatives of birds, have lost their clavicles during evolution and retained the interclavicle as the sole dermal bone element of the shoulder girdle (Fig. 1D and fig. S1J). In *Caiman yacare*, we observed paired interclavicular ossification centers with a comma-shaped morphology (Fig. 2K) (*21*), consistent with previous descriptions for *Alligator mississippiensis* (*8*, *22*). These centers subsequently elongate and fuse in a cranial-to-caudal direction (*8*).

To further assess the homology of the furcula, we analyzed additional reptiles in which clavicles are present. In geckos, observations from *Paroedura picta* and *Eublepharis macularius* together indicate that clavicles begin ossifying from elongated centers in the lateral third of each element (Fig. 2G). Shortly thereafter, two thin interclavicular ossification centers appear near the midline. These are craniocaudally elongated, closely opposed, and positioned immediately caudal to the clavicle (Fig. 2H). *Iguana iguana* displays a similar pattern of the clavicular ossification, although interclavicular centers appear markedly delayed and were not observed in detail (fig. S1, G and H).

In turtles, the clavicles and the interclavicle are integrated into the plastron (*8*, *9*, *23*). However, a comparable ossification pattern was observed in *Trachemys scripta*: The developing epiplastra (homologous to the clavicles) ossify first as elongated lateral centers, whereas the entoplastron (homologous to the interclavicle) was observed as a single ossification center with a distinctly bilobed morphology, likely reflecting rapid fusion of initially paired centers (Fig. 2L).

The comparison of dermal ossification in the reptilian shoulder girdle reveals a conserved pattern. When present, the clavicles always begin ossifying earlier than the interclavicle, appearing in a craniolateral position and later elongating toward the midline without ever contacting each other. The interclavicle, in turn, originates as either single or paired centers that fuse along the midline in a cranial-to-caudal sequence, forming a single element with bilateral morphology.

### Preosteogenic pattern of the dermal shoulder girdle

Before ossification, dermal bones form through mesenchymal cell aggregation into preosteogenic condensations. Because these condensations represent the initial step in bone formation, their analysis provides a more direct inference of ancestral bone presence (*18*, *24*, *25*). Previous work has shown that both the embryonic interclavicle of *A. mississippiensis* and the furcula of *G. gallus* originate from paired preosteogenic mesenchymal condensations that fuse at the midline to form a single bone (*8*). However, the detailed 3D pattern of these condensations remains unknown, particularly in key groups such as *Nothoprocta*, whose furcula ossifies from three distinct centers. We therefore investigated the pattern of mesenchymal condensation formation to determine whether the observed ossification centers correspond to independent primordial structures. We used an approach combining histology with 3D reconstructions, which allowed us to assess both the histological and cellular detail of the condensations, as well as their overall topology and morphology (Fig. 3).

**Fig. 3.**
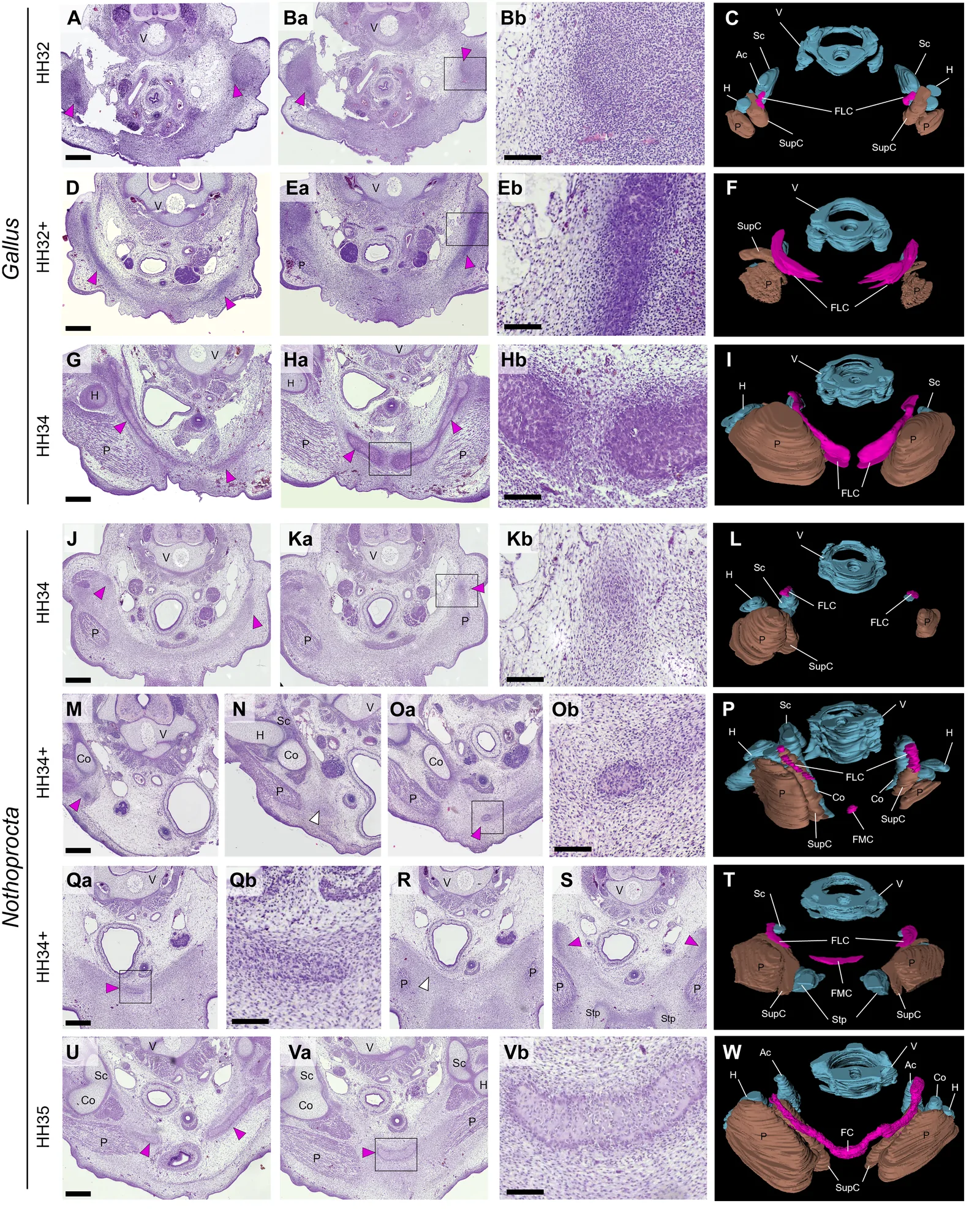
Histological details of furcular condensations and 3D reconstructions in *Gallus* and *Nothoprocta*. Each row corresponds to a single specimen. (**A** to **C**) HH32 *Gallus* embryo. (**D** to **F**) HH32+ *Gallus* embryo. (**G** to **I**) HH34 *Gallus* embryo. (**J** to **L**) HH34 *Nothoprocta* embryo. (**M** to **P**) HH34+ *Nothoprocta* embryo. (**Q** to **T**) HH34+ *Nothoprocta* embryo. (**U** to **W**) HH35 *Nothoprocta* embryo. (A), (Ba), (D), (Ea), (G), (Ha), (J), (Ka), (M), (N), (Oa), (Qa), (R), (S), (U), and (Va) are histological transverse sections sequentially ordered from cranial to caudal, shown left to right, for each specimen. Scale bars, 300 μm. (Bb), (Eb), (Hb), (Kb), (Ob), (Qb), and (Vb) are higher magnifications of (Ba), (Ea), (Ha), (Ka), (Oa), (Qa), and (Va), respectively, showing cellular detail of the preosteogenic mesenchymal condensations of the furcula. Scale bars, 100 μm. Magenta arrowheads indicate the mesenchymal condensations of the furcula. White arrowheads in (N) and (R) indicate the absence of condensed mesenchyme, demonstrating discontinuity between the lateral condensations and the medial condensation of the furcula in *Nothoprocta*. (C), (F), (I), (L), (P), (T), and (W) are 3D reconstructions of the corresponding specimens. The furcular condensations (magenta), cartilaginous skeleton (sky blue), and pectoral musculature (pale brown) are shown to aid anatomical interpretation. Labeled anatomical elements: Ac, acromion; Co, coracoid; FC, fused furcular condensation; FLC, furcular lateral condensation; FMC, furcular medial condensation; H, humerus; P, pectoralis muscle; Sc, scapula; Stp, sternal plates; SupC, supracoracoideus muscle; V, vertebrae.

We analyzed the preosteogenic development in *Gallus* and *Nothoprocta* embryos. Initial steps of furcula development are very similar in both species, although slightly delayed in *Nothoprocta*. The first discrete elements attributable to the furcula are a pair of cellular preosteogenic condensations located cranioventral to the scapular acromion, composed of densely packed cells with larger nuclei and a modest amount of ECM. This condition was observed in *Gallus* at stage HH32 and *Nothoprocta* at stage HH34 (Fig. 3, A to C and J to L).

Subsequently, in *Gallus*, both lateral condensations grow toward the midline (Fig. 3, B to F), resembling the pattern observed during ossification. Medial growth of the condensations continues until they are closely contiguous at the midline but remain unfused (Fig. 3, G to I). At this stage, the condensations are in an osteoid phase, with a broad core of eosinophilic matrix surrounded by osteoblasts with a few scattered cells visible inside the matrix core (Fig. 3Hb). The fusion of condensations occurs at stage HH35 (fig. S3A), but separation is still evident in ossification until stage HH36 (fig. S3, B to D).

In *Nothoprocta* at the advanced stage HH34 (HH34+), a third furcular preosteogenic condensation at a medial position becomes evident (Fig. 3, M to T). The three condensations were topologically consistent with the ossification centers described above: two lateral and one medial (Figs. 2E and 3, P and T). The discontinuity between the medial and lateral condensations was evident when tracing them through the stacked slices and was confirmed in the 3D reconstruction (Fig. 3, P and T). Thereafter, the three condensations fuse rapidly, and by stage HH35, the furcula appears as a single continuous osteoid phase condensation without signs of ossification (Fig. 3, U to W).

We used the same approach to observe the early morphogenesis of clavicles and interclavicles in non-avian reptiles. In *Caiman*, a paired interclavicular condensation was formed by a dense aggregation of mesenchymal cells (Fig. 4, A to D). Condensations were craniocaudally elongated and positioned close to each other near the midline. In *Trachemys* and *Iguana*, we detected the developing shoulder dermal elements at a more advanced developmental stage, in the osteoid phase (Fig. 4, E to P). In both species, clavicular condensations develop near the acromion process of the scapula, prior to any evidence of a developing interclavicle. In *Trachemys*, the interclavicle develops later as a single medial condensation (Fig. 4, F and H), whereas in *Iguana*, interclavicular condensations were not detected at the stages examined.

**Fig. 4.**
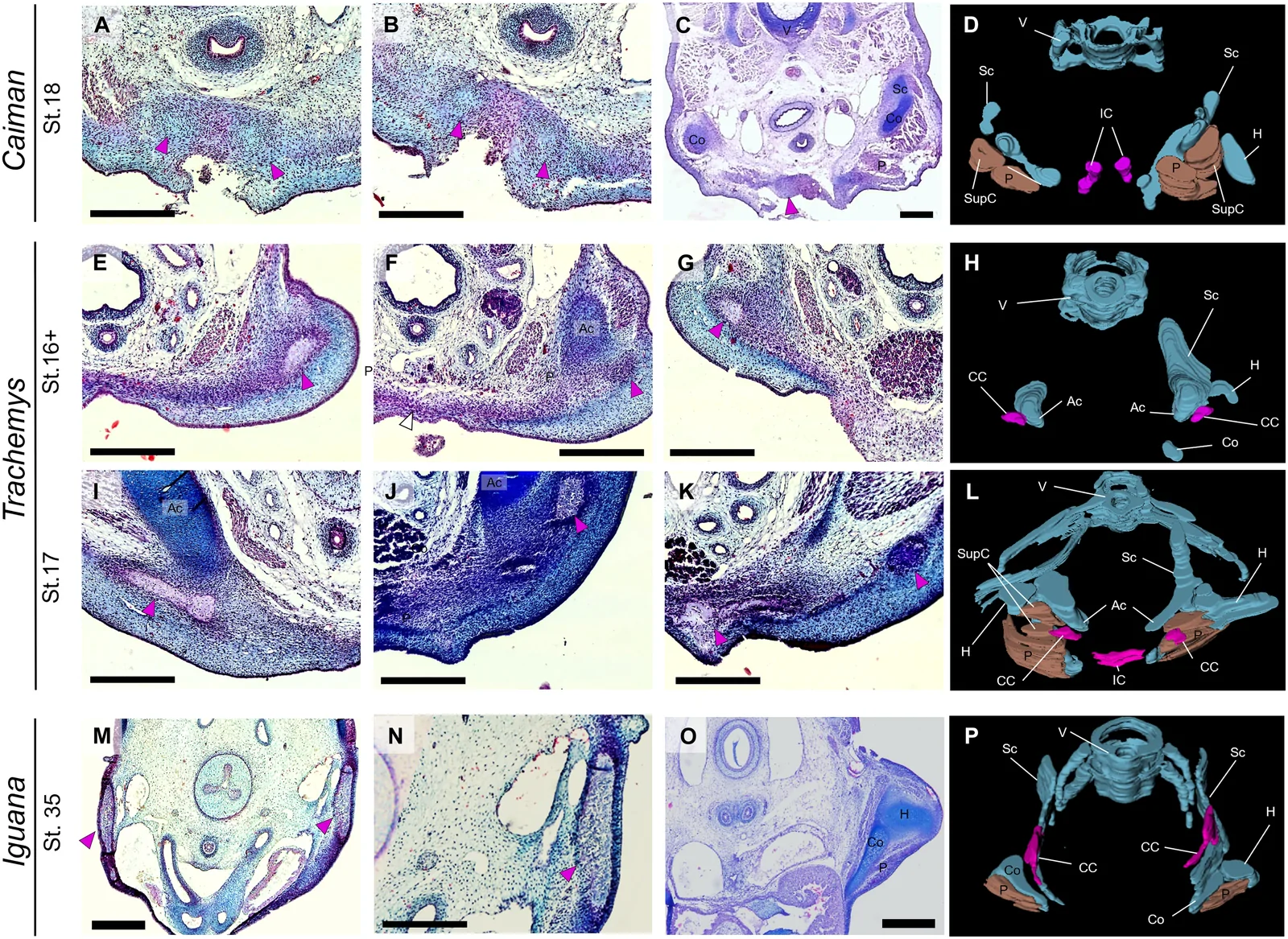
Histological detail of dermal shoulder girdle condensations and 3D reconstructions in *Caiman*, *Trachemys*, and *Iguana*. (**A** to **D**) Stage 18 *Caiman yacare* embryo. (**E** to **H**) Stage 16+ *T. scripta* embryo. (**I** to **L**) Stage 17 *Trachemys* embryo. (**M** to **P**) Stage 35 *I. iguana* embryo. (A) to (C), (E) to (G), (I) to (K), and (M) to (O) are histological transverse sections sequentially ordered from cranial to caudal, shown left to right, for each specimen. Scale bars, 300 μm. Magenta arrowheads indicate mesenchymal condensations of the interclavicle (medial) and clavicles (lateral). The white arrowhead in (F) indicates the absence of an interclavicular condensation at the midline in *Trachemys* late stage 16. (D), (H), (L), and (P) are 3D reconstructions of the same specimens, showing interclavicular and clavicular condensations (magenta), cartilaginous skeleton (sky blue), and pectoral musculature (pale brown). Labeled anatomical elements: CC, clavicular condensation; IC, interclavicular condensation.

## DISCUSSION

The conventional view that the furcula originated by the medial fusion of clavicles has been recently challenged by embryological and paleontological evidence (*7*, *13*, *26*). While recent observations suggest that its reinterpretation as an interclavicle is at least equally parsimonious, few discussed traits are actually exclusive to the interclavicle, and the involvement of the clavicles has not been ruled out. Rather than supporting either of these mutually exclusive hypotheses, our embryological data indicate that the furcula incorporates both clavicular and interclavicular components.

In both *Gallus* and *Nothoprocta*, the lateral furcular preosteogenic condensations arise in close proximity to the scapular acromion and subsequently extend medially, a developmental pattern mirrored in ossification and closely matching the clavicular development observed in turtles and lepidosaurs. Together, these conserved topological relationships strongly support the homology between the lateral components of the furcula and the clavicles.

Turtles provide a particularly valuable comparison because they are the closest extant lineage to birds retaining both clavicles and an interclavicle (*23*, *27*). This comparison allowed us to show that both the clavicles and the lateral furcular condensations arise in a similar topological position adjacent to the acromion, even in turtles, where the pectoral girdle has undergone extensive evolutionary rearrangement and the acromion process is extremely elongated.

Our results provide direct embryological evidence for the formation of an interclavicle in a paleognathous bird, the Chilean tinamou *Nothoprocta*. The discovery of an ossification center for the interclavicle is correlated with its early formation as an independent preosteogenic condensation, separate from the clavicular condensations that become the arms of the furcula (Fig. 3, P and T). We have discarded the possibility that a single large preosteogenic condensation is formed, that later develops three ossification centers, or that only the clavicular condensations are formed, later developing a third ossification center at their medial site of fusion. These alternative scenarios would have limited the evidence for an ancestral skeletal element to an ossification pattern, with no additional support from early condensation stages. Comparisons with relevant non-avian taxa (with an interclavicle) also confirm the medial condensation of *Nothoprocta* forms and ossifies in the expected position and pattern of the interclavicle. *Nothoprocta* belongs to Palaeognathae, the sister lineage to Neognathae, to which all other studied species with a fully formed furcula belong. This makes *Nothoprocta* a key taxon for evaluating hypotheses on the evolutionary origin of the furcula. The phylogenetic distribution of the developmental pattern in reptiles and birds is consistent with the medial condensation representing the ancestral interclavicle, retained in *Nothoprocta*, and subsequently lost in neognaths, rather than a neomorphic element independently acquired in this lineage (Fig. 5A).

**Fig. 5.**
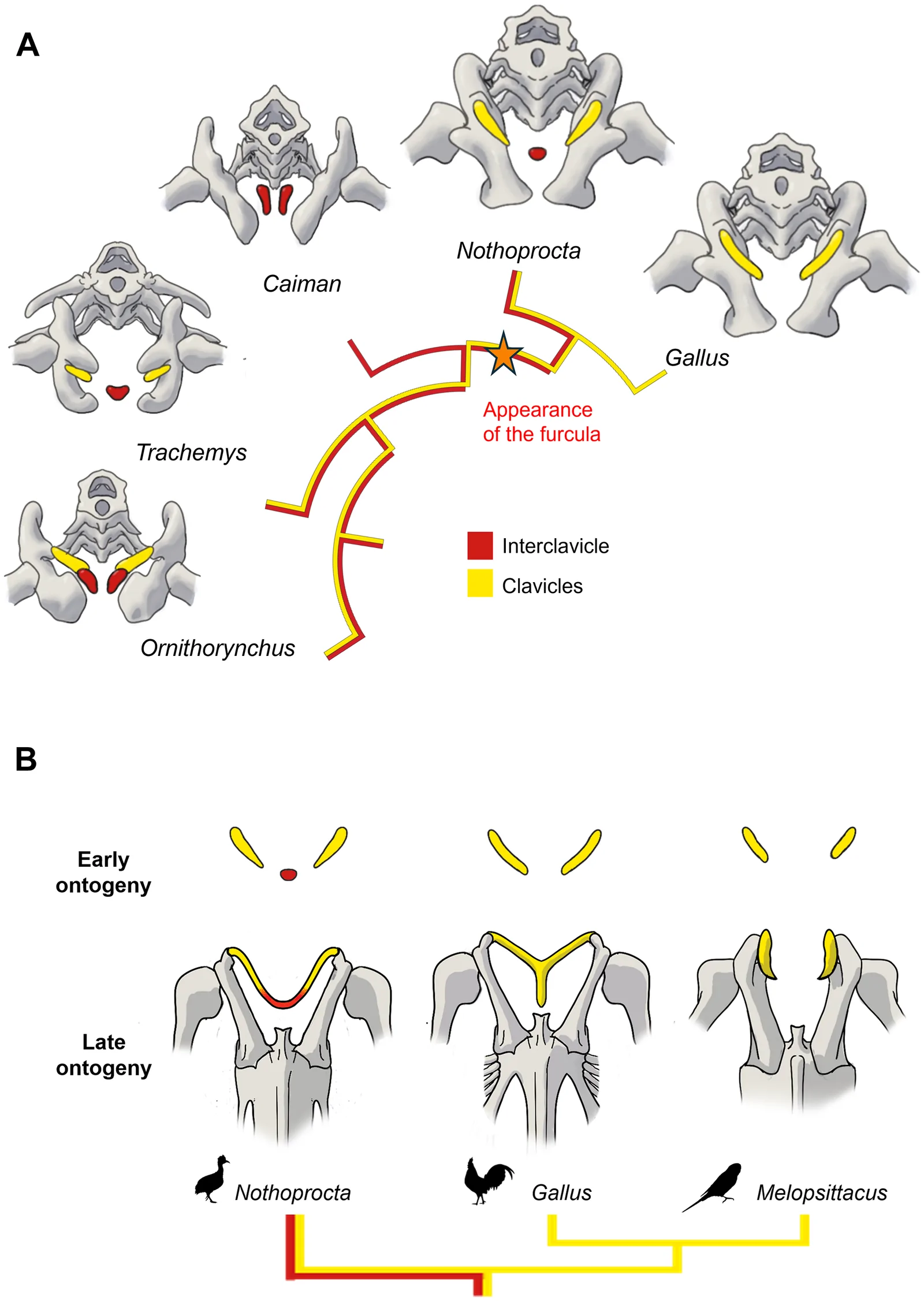
Evolution of the developmental pattern of the dermal shoulder girdle in amniotes and its evolutionary consequences. (**A**) Comparative schematic model summarizing how the pattern of early preosteogenic condensations evolved across amniotes. Schematic representations of *Trachemys*, *Caiman*, and *Nothoprocta* are based on developmental reconstructions obtained in this study; the *Ornithorhynchus* schematic is based on Klima, 1973 (*44*). The distribution of clavicular (yellow) and interclavicular (red) condensations illustrates the conservation of the ancestral pattern in palaeognaths and its secondary simplification in neognaths and reveals the composite origin of the avian furcula. (**B**) Hypothesized developmental pathway leading to the evolution of clavicle-like furculae. In *Nothoprocta*, and inferred ancestrally for birds, the furcula develops from paired clavicular condensations and a medial interclavicular condensation. In Neognathae (e.g., *Gallus*), the interclavicular component is lost, but the furcula still forms by medial extension and fusion of the clavicular condensations. In some birds, the adult furcula is “deconstructed” into short, paired clavicle-like elements. We propose that loss of the interclavicular condensation preceded and enabled the evolution of a deconstructed furcula.

The development of the furcula in *Nothoprocta* also shows the early interclavicle condensation fuses to the paired condensations of the clavicles, demonstrating the composite nature of the furcula. This readily explains why both the interclavicle or clavicle hypotheses have so far been unable to exclude each other. The composite origin by fusion of these structures may help explain why the fossil record does not document any evolutionary process in which one of these elements is retained, while the other becomes reduced and disappears. Transitional fossil taxa with visible sutures within the furcula could have potentially revealed its composite nature, but the absence of any such evidence suggests that the furcula did not start out developing by late fusion of separate bony elements but from the early fusion of preosteogenic condensations—as seen in *Nothoprocta*.

The furcula originated early in theropod evolution, well before the emergence of powered flight, which therefore is not the functional context of its origin. Toward the origin of Theropoda, it is hard to identify the emergence of a new function that could require the fusion of the interclavicle with the clavicles. Rather, the most conspicuous change in their functional context is the great reduction in size of these elements, which has greatly complicated their preservation and fossil record in early dinosaurs. Especially in theropods, this reduction may relate to the small size and relaxed functional commitment of the forelimbs within increasingly specialized bipedal-cursorial locomotion and increased emphasis on the head and a slender neck for predation/foraging—functions that are still carried out by birds with little or no direct aid of the forelimbs. In the early theropod *Coelophysis*, proportionally small forelimbs and furcula (only slightly larger than those of *Archaeopteryx* and *Gallus* despite its much larger body size) were accompanied by unusually high intraspecific morphological variation of the forelimbs, a documented correlate of relaxed functional commitment (*5*, *7*, *28*). In many non-avian theropod lineages, the small size of the furcula relative to body and forelimb size may have limited its mechanical role, possibly restricting it to a simple strut between the scapulocoracoid articulation. The relative reduction of this component of the pectoral girdle may also have facilitated the early coalescence of the interclavicular and clavicular condensations, as bone fusion is sometimes associated with the evolutionary size reduction of skeletal elements (*29*, *30*). Given that reduction is the most conspicuous change at around the time the clavicle and interclavicle fused, we suggest that it may have led to their fusion, without necessarily requiring selection for a new function.

The fusion of these small, functionally reduced structures, however, may have enabled important functional transformations later in evolution. In ornithuromorphs, the furcula became relatively larger and more U-shaped, in association with the elongation of the coracoids and the dorsal displacement of the glenoid fossae. In several lineages, a medial connection with the sternum was re-established through the hypocleidium, ligaments, or even direct fusion at the medial interclavicular region (*5*, *31*–*33*). This new configuration allowed the furcula to contribute more substantially to the stabilization of the shoulder girdle and, through elastic deformation, to act as a spring during the wingbeat cycle (*34*, *35*). The earlier fusion of the clavicular and interclavicular components, occurring under relaxed functional constraints, may therefore have facilitated the subsequent acquisition of this new mechanical role. As Charles Darwin observed, “An organ may become rudimentary for its proper purpose, and be used for a distinct object” (*36*).

The composite nature of the furcula can also be obscured if we only obtain embryological data from a limited sample of model taxa. Although the chicken *G. gallus* is a popular model taxon for research—and often, the only source of information about any bird—it has experienced evolutionary change in the development of the furcula, without any clear effects on the adult structure. In *Gallus*, we found no embryological evidence of an interclavicle: the furcula develops exclusively from paired clavicles, with a visible gap at the comparable embryological position in *Nothoprocta* where the interclavicle condensation is formed (fig. S3). The clavicular condensations of *Gallus* then extend medially and fuse at the midline, where no medial ossification center is formed. In the light of the ancestral pattern of *Nothoprocta*, we can conclude that the development of *Gallus* furcula is best interpreted as a secondarily acquired simplification (Fig. 5A). In many other bird lineages, including *Melopsittacus*, the furcula has ceased to be a single element and has become deconstructed into separate paired elements set above the coracoids, resembling the clavicles. We suggest that a previous step enabling the evolution of this derived condition is the loss of the interclavicular preosteogenic condensation, as observed in *Gallus* (Fig. 5B). This proposed sequence can reflect how some changes in developmental pathways may have no immediate phenotypic effects (*37*, *38*) but nevertheless still have consequences for future possibilities of evolutionary change (*39*).

## MATERIALS AND METHODS

### Embryo collection and staging

Fertile eggs of broiler chicken (*G. gallus*) were obtained from Avícola Chorombo (Santiago, Chile) on the same day they were laid. Fertile eggs of Chilean tinamou (*N. perdicaria*) were obtained from the commercial breeder “Tinamou Chile” (Los Ángeles, Chile). For budgerigar (*M. undulatus*) and zebra finch (*T. guttata*), eggs were obtained from colonies maintained at the Ontogeny and Phylogeny Laboratory, University of Chile (Santiago, Chile). Eggs were incubated in an automatic incubator with rotating trays at 37°C and 60% humidity. The removed embryos were washed in 1× phosphate-buffered saline and fixed directly in methanol for wholemount skeletal staining or in 4% paraformaldehyde (PFA) for 24 hours for histology. PFA-fixed embryos were dehydrated in a methanol series and stored at −20°C. Embryonic stages were standardized according to the HH table of normal development (*19*).

*P. picta* embryos fixed in PFA for 24 hours and stored in methanol were donated by the Department of Geology and Geophysics at Yale University (New Haven, Connecticut, USA), while *E. macularius* embryos were removed from eggs of our colony and incubated at 30°C and 90% humidity (ZOOMED reptibator incubator). Embryos were staged using the species-specific developmental tables (*40*, *41*).

PFA fixed embryos stored in methanol of *C. yacare*, *T. scripta*, and *I. iguana* were obtained from the Herpetological Collection of Ribeirão Preto from University of São Paulo (CHRP-USP, Ribeirão Preto, São Paulo, Brazil). Their embryonic stages were standardized using specific developmental tables (*21*, *42*, *43*).

### Whole mount single alizarin red stain

Embryos stored in methanol were immersed in 0.02% alizarin red (Sigma-Aldrich) with 0.5% KOH aqueous solution for 2 hours. Excess dye was removed by washing with distilled water, followed by clearing in 0.5% KOH solution and then successively incubated in a glycerol series until 85% glycerol for storage. A previous treatment of 20 to 30 min of maceration in trypsin 1% in 70% distilled water 30% saturated borate solution was applied for PFA-fixed specimens.

### Whole mount double skeletal staining

Embryos stored in methanol were immersed for 2 to 3 days in 0.05% Alcian blue 8GX (Sigma-Aldrich) prepared in 80% ethanol and 20% glacial acetic acid. The excess dye was removed by washing three times for 10 min in 70% ethanol. After rehydration, specimens were incubated for 2 hours in 0.02% alizarin red S (Sigma-Aldrich) in 0.5% KOH. Excess alizarin was removed by two washes in distilled water, followed by clearing in 0.5% KOH. Specimens were then transferred to 20% glycerol in 0.5% KOH and successively incubated in a glycerol series until 85% glycerol for storage.

### Histology and 3D reconstruction

Embryos fixed in PFA and stored in methanol were embedded in paraffin. Sections were cut with a microtome to a thickness of 4 or 5 μm and mounted on slides treated with silane. Paraffin sections were stained with hematoxylin and eosin, hematoxylin-eosin-Alcian blue, Masson’s trichrome, or Mallory’s trichrome following standard histological protocols. Histological sections of *Gallus* and *Nothoprocta* were scanned using a Motic EasyScan system. For *Caiman*, *Trachemys*, and *Iguana*, serial sections were photographed using a stereomicroscope equipped with a digital camera. Section images were exported as JPG files and imported into FIJI (“Fiji Is Just ImageJ”) as image stacks. For 3D reconstruction, image stacks were generated using every second section, resulting in a final section spacing of 8 or 10 μm. The TrackEM2 plugin was used to generate an automatic alignment that was then corrected manually. The resulting stacks were analyzed in 3D Slicer, where stack spacing was adjusted, and the volume was segmented to obtain a 3D reconstruction of the furcula condensations, cartilaginous structures, and pectoral musculature. The pectoral musculature was included to provide anatomical landmarks for orienting the furcular condensations relative to the shoulder girdle.

### Ethics statement

All procedures involving animals were approved by the Institutional Animal Care and Use Committee of the University of Chile (protocol 19267-FCS-UCH) and by the Scientific Ethics Committee for the Care of Animals and the Environment of the Pontificia Universidad Católica de Chile (protocol 181107003).
