# Supplementary material for: Embryos resolve the enigmatic origin of the theropod wishbone
Source: Sci Adv. 2026 Oct 7;12(41):eaeh7555. doi: 10.1126/sciadv.aeh7555 (PMC13644770; doi:10.1126/sciadv.aeh7555)
Supplement: Supplementary file 1 [file sciadv.aeh7555_sm.pdf]

Science Advances
AAAS

Supplementary Materials for

Embryos resolve the enigmatic origin of the theropod wishbone

Sebastian Jiménez-Villalón *et al.*

Corresponding author: Sebastian Jiménez-Villalón, sebastian.jimenez@ug.uchile.cl; Joao F. Botelho, chicobotelho@gmail.com; Alexander O. Vargas, alexvargas@uchile.cl

Sci. Adv. **12**, eaeh7555 (2026)
DOI: 10.1126/sciadv.aeh7555

This PDF file includes:

Figs. S1 to S3

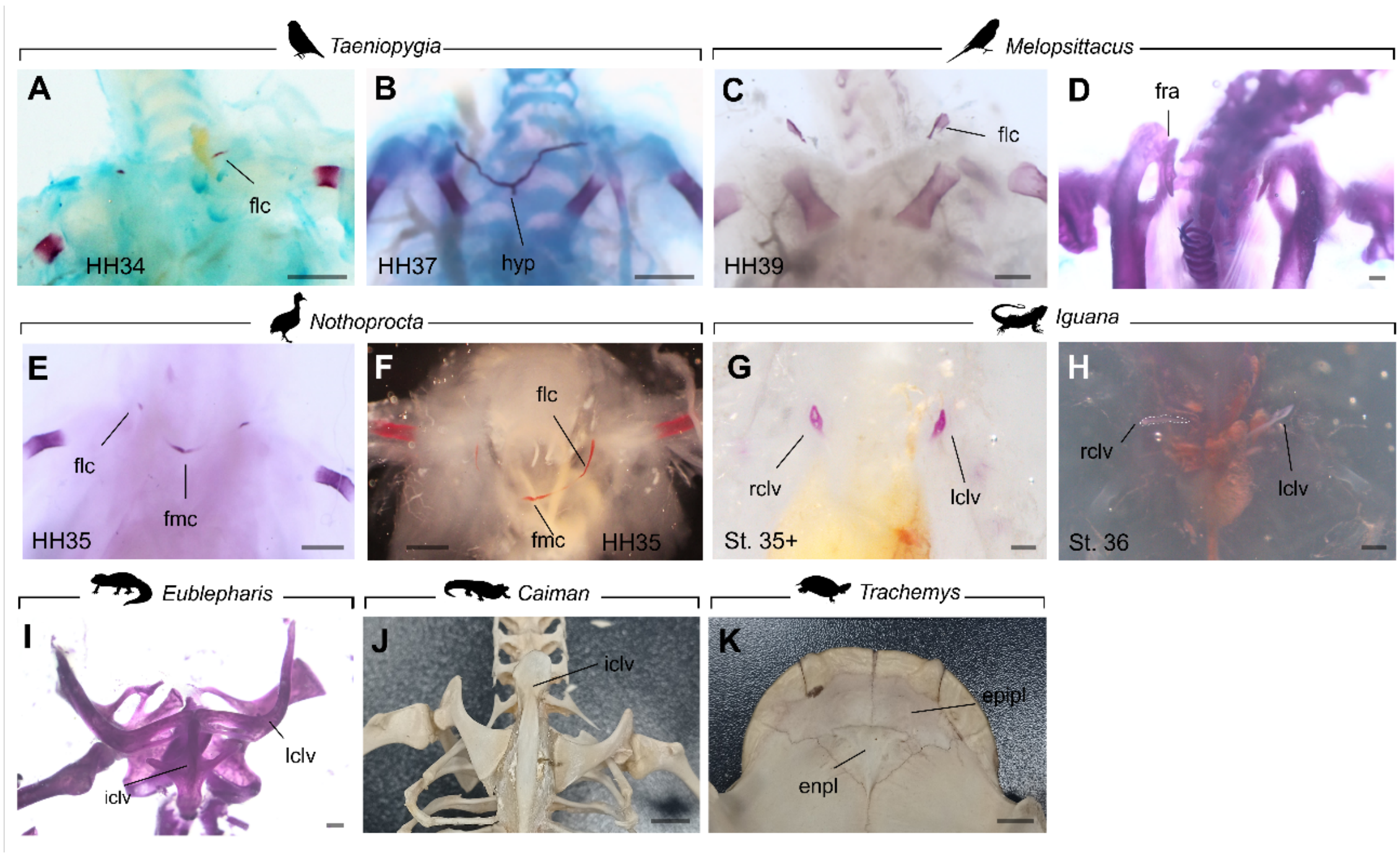

Figure S1. Additional specimens showing dermal shoulder girdle ossification in birds and non-avian reptiles. (A-B) Alizarin red and Alcian blue whole-mount stained preparations; (C-I) Alizarin red whole-mount stained preparations. (A) HH34 *Taeniopygia* embryo showing early furcular ossification. (B) HH37 *Taeniopygia* embryo showing a well-developed hypocleidium. (C) HH39 *Melopsittacus* embryo showing lateral furcular centers having reached their full extent. (D) Adult *Melopsittacus* displaying separate furcular arms. (E, F) additional specimens of HH35 *Nothoprocta* embryos showing the presence of three distinct furcular ossification centers. (G) Stage 35+ *Iguana* embryo showing the early ossification centers of the clavicles. (H) Stage 36 *Iguana* embryo showing further medially elongated clavicular centers. (I) Adult *Eublepharis* showing the interclavicle and clavicles. (J) Skeletal preparation of juvenile *Caiman* showing the interclavicle. (K) Skeletal preparation of an adult *Trachemys* plastron in dorsal view showing epiplastra (clavicles) and entoplastron (interclavicle). Labeled anatomical elements: flc = furcular lateral center; fra = furcular right arm; hyp = hypocleidium; rclv = right clavicle; lclv = left clavicle; fmc = furcular medial center; iclv = interclavicle; epipl = epiplastron; enp = entoplastron. Scale bars: 1 mm (A-I); 1 cm (J-K).

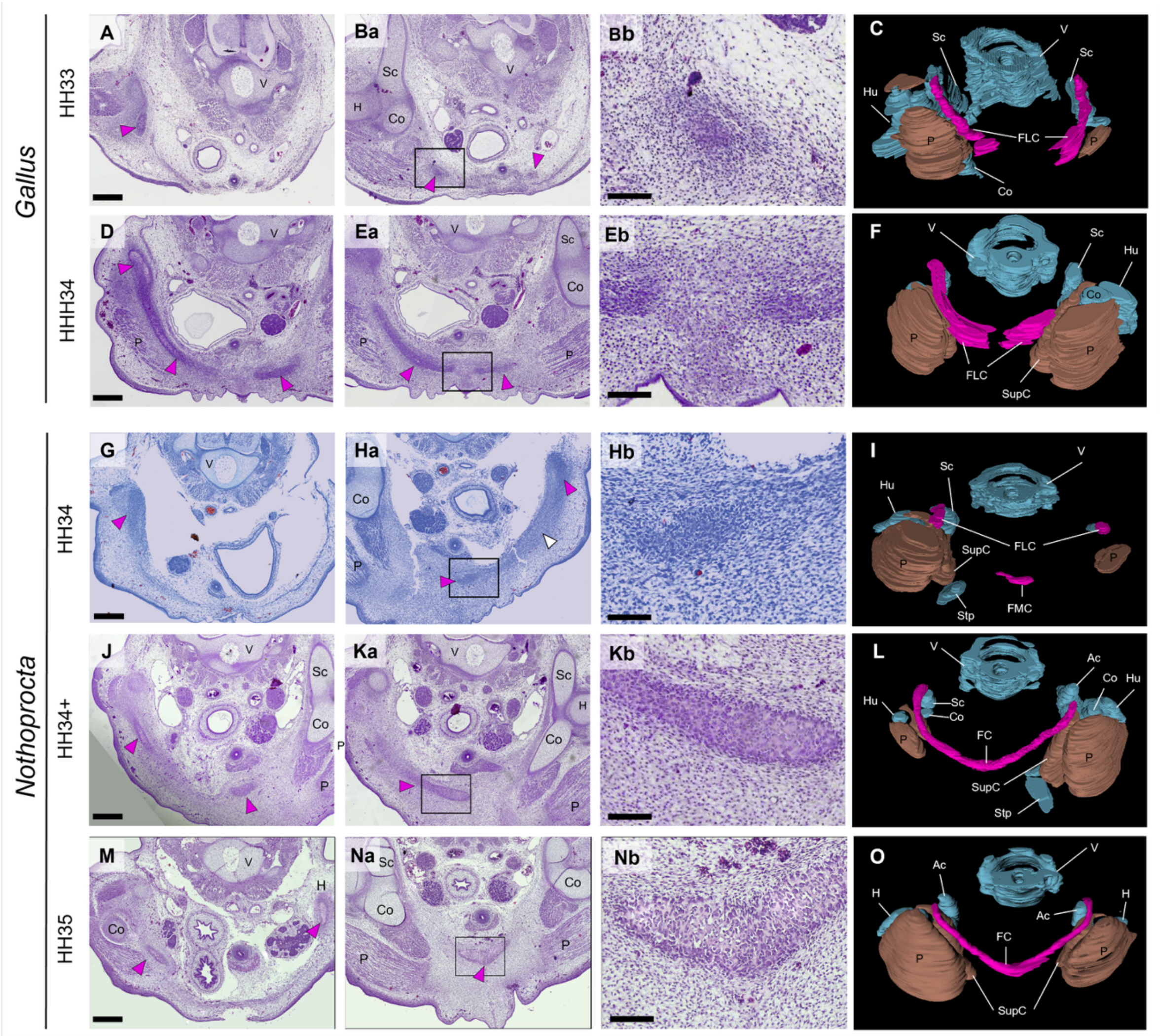

Figure S2. Additional specimens of *Gallus* and *Nothoprocta* showing the histological details of furcular condensations and 3D reconstructions. Each row corresponds to a single specimen. (A-C) HH33 *Gallus* embryo; (D-F) HH34 *Gallus* embryo; (G-I) HH34+ *Nothoprocta* embryo; (J-L) HH34+ *Nothoprocta* embryo; and (M-O) HH35 *Nothoprocta* embryo. (A, B) Histological transverse sections sequentially ordered from cranial to caudal, and (C) 3D reconstruction of the corresponding specimen; (D, E) histological sections and (F) 3D reconstruction; (G, H) histological sections and (I) 3D reconstruction; (J, K) histological sections and (L) 3D reconstruction; (M, N) histological sections and (O) 3D reconstruction. Histological sections are shown from cranial to caudal, left to right (scale bar: 300 µm). (A, Ba, D, Ea, J, Ka, M, Na) are stained with H&E, and (G, Ha) with Mallory's trichrome. (Bb, Eb, Hb, Kb, Nb) are higher magnifications showing cellular detail of the preosteogenic mesenchymal condensations (scale bar: 100 µm). Magenta arrowheads indicate furcular condensations; white arrowheads in Ha indicate the absence of condensed mesenchyme between the lateral and medial condensations. The 3D reconstructions show furcular condensations (magenta), cartilaginous skeleton (sky blue), and pectoral musculature (pale brown). Labeled anatomical elements: Ac = acromion; Co =

coracoid; FC = fused furcular condensation; FLC = furcular lateral condensation; FMC = furcular medial condensation; H = humerus; P = pectoralis muscle; Sc = scapula; Stp = sternal plates; SupC = supracoracoideus muscle; V = vertebrae.

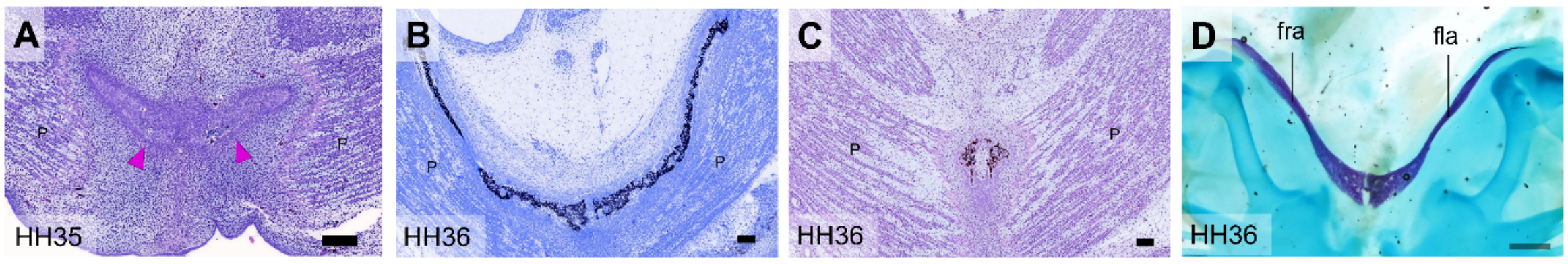

Figure S3. Additional specimens of *Gallus* show the persistent separation between the two furcula arms. (A) HH35 *Gallus* embryo with fusion of both lateral condensations. (B) HH36 *Gallus* transverse section stained with Mallory's trichrome and Von Kossa showing the gap between the ossification centers. (C) Same specimen as in (B), a more caudal section stained with H&E, Alcian Blue and Von Kossa showing the gap. (D) Alizarin red and Alcian blue double stained whole-mount *Gallus* HH36 embryo. Scale bars in A, B and C: 300 µm. Labeled anatomical elements: fra = furcular right arm; fla = furcular left arm; P = pectoralis muscle.
